# Prognostic Value of a New Marker of Ventricular Repolarization in Cirrhotic Patients

**DOI:** 10.5935/abc.20160181

**Published:** 2016-12

**Authors:** Angelo Antunes Salgado, Paulo Roberto Benchimol Barbosa, Alinne Gimenez Ferreira, Camila Aparecida de Souza Segrégio Reis, Carlos Terra

**Affiliations:** Hospital Universitário Pedro Ernesto - Universidade Estadual do Rio de Janeiro (UERJ), RJ - Brazil

**Keywords:** Liver Cirrhosis / etiology, Liver Cirrhosis / mortality, Cardiomyopathies / physiopathology, Electrocardiography, Prognosis, Ventricular Dysfunction, Left / physiopathology

## Abstract

**Background:**

There is still debate about the relationship between changes in ventricular repolarization on the surface electrocardiogram and cirrhosis severity.

**Objective:**

To study the relationship between variables related to ventricular repolarization and the clinical severity of the cirrhotic disease.

**Methods:**

We selected 79 individuals with hepatic cirrhosis, classified according to the Child-Pugh-Turcotte criteria (Child A, B, and C). We measured the QT and corrected QT (QTc) intervals, and the interval between the peak and the end of the T wave (TpTe), and we identified their minimum, maximum, and mean values in the 12-lead electrocardiogram. We also calculated the dispersion of the QT (DQT) and QTc (DQTc) intervals.

**Results:**

In 12 months of clinical follow-up, nine subjects underwent hepatic transplantation (Child A: 0 [0%]; Child B: 6 [23.1%]; Child C: 3 [18.8%]; p = 0.04) and 12 died (Child A: 3 [12.0%]; Child B: 4 [15.4%]; Child C: 5 [31.3%]; p = 0.002). No significant differences were observed between the cirrhotic groups related to the minimum, maximum, and mean values for the QT, QTc, TpTe, DQT, and DQTc intervals. A minimum TpTe interval ≤ 50 ms was a predictor for the composite endpoints of death or liver transplantation with a sensitivity of 90% and a specificity of 57% (p = 0.005). In the Cox multivariate analysis, the Child groups and a minimum TpTe of ≤ 50 ms were independent predictors of the composite endpoints.

**Conclusion:**

The intervals QT, QTc, DQT, DQTc, and TpTe have similar distributions between different severity stages in cirrhotic disease. The TpTe interval proved to be a prognostic marker in subjects with cirrhosis, regardless of disease severity (NCT01433848).

## Introduction

Liver cirrhosis is defined as a diffuse disorganization of the hepatic architecture resulting from chronic aggression to the liver, producing fibrosis and regenerative nodules. Fibrosis is potentially reversible if the cause of the aggression is removed, but it becomes irreversible in advanced stages when significant vascular and cellular changes are observed.^1^

Cirrhotic cardiomyopathy is a chronic cardiac dysfunction in cirrhotic patients, characterized by an inadequate contractile response to stress, changes in diastolic function and/or electrophysiological abnormalities in the absence of known heart disease.^2,3^ Studies in animal models have demonstrated that cirrhotic cardiomyopathy involves multiple mechanisms, such as changes in the ion channel membrane of the cardiomyocytes, a reduction in the density and functionality of beta-adrenergic receptors, impairment of the contractile protein structure of the extracellular matrix, and an increase in the interstitial concentration of substances with vasodilatory and pro-apoptotic effects, such as nitric oxide, proinflammatory cytokines, and endogenous cannabinoids.^4^

The electrical abnormalities commonly described include changes in the QT and corrected QT (QTc) intervals, associated with the evolutionary stages of the cirrhosis.^5^ It has been suggested that the QT interval may represent a prognostic marker of autonomic dysfunction and survival in cirrhotic cardiomyopathy.^5^ In spite of studies correlating mechanical changes (such as systolic and diastolic dysfunction) with electrical changes that characterize the cirrhotic cardiomyopathy, the prognosis of patients with liver cirrhosis and long QT is still unclear, and the results associated with mortality remain controversial.^6-8^

Several hypotheses have been raised to justify the apparent lack of definition between results from different studies. Among them, we highlight the heterogeneity of samples, a lack of uniformity in methodologies, as well as different clinical follow-up durations. In contrast, new electrical markers, including the interval between the peak and the end of the T wave (TpTe), stand out due to their ability to infer the presence of refractory transmural dispersion, with potential application in the stratification of arrhythmogenic risk in different populations.^9-11^ However, little is known about the behavior of this marker in cirrhotic stages.

Thus, considering the clinical implications of cirrhotic cardiomyopathy, this study aimed to evaluate a set of measures for the ventricular repolarization on the surface electrocardiogram (ECG) and to relate these measures with the various stages of clinical disease severity estimated by the Child-Pugh-Turcotte classification.^12^

## Methods

### Study population

This was a prospective and observational study, conducted in a reference center of the *Sistema Único de Saúde* (Brazilian Unified Health Care System [SUS]) for the treatment of liver disease, with the inclusion of 79 individuals aged between 18 and 80 years and of both sexes, stratified according to the Child-Pugh-Turcotte classification (classes A, B and C). The inclusion criteria were: i) liver cirrhosis confirmed by an imaging method (ultrasound/magnetic resonance), laboratory tests, upper digestive endoscopy, and/or liver biopsy; and ii) sinus rhythm. The exclusion criteria were: i) hypertension in treatment; ii) congestive heart failure; iii) ischemic, valvular, or any other heart disease; iv) chronic obstructive pulmonary disease; v) peripheral artery disease; vi) infection or recent bleeding (in the last two weeks prior to the potential consideration for inclusion in the study); vii) anemia characterized by hemoglobin levels < 9 g/dL; viii) type 1 diabetes mellitus; ix) chronic kidney disease; x) transjugular intrahepatic portosystemic shunt (TIPS); xi) pregnancy; or xii) use of illicit drugs. Beta-blockers were maintained because of the risk of bleeding due to portal hypertension.

The Child-Pugh-Turcotte classification is a classic index of mortality in cirrhotic patients, initially used to define the prognosis of these patients when undergoing surgery to treat portal hypertension. Since then, the index has been used as a prognostic reference for cirrhotic patients in general. This index includes three laboratory variables (serum measurement of bilirubin, albumin, and prothrombin time) and two clinical variables (ascites and encephalopathy), to which a scale of points is assigned, classifying the patients according to their cirrhotic disease stage as early (Child A), intermediate (Child B), and advanced (Child C).

The subjects were followed up as outpatients for up to 12 months, in visits scheduled every 3 months. During the follow-up, the following endpoints were assessed: i) death, and ii) death or liver transplant. The study protocol was approved by the research ethics committee at the Pedro Ernesto University Hospital - UERJ (CEP/HUPE 2857-2010) and included in the Clinicaltrials.gov database under the registry NCT01433848. An informed consent form was obtained from all subjects.

### Study protocol

We analyzed 12-lead ECGs obtained at rest, printed on graph paper at a speed of 25 mm/s, and properly calibrated with 1 mv = 10 mm (N). The tests were performed in the morning with the subjects lying down comfortably in the supine position. The following standards were used to assess the quality of the tracings: i) quality of the tracing printout; ii) T wave poorly visualized, preventing correct measurement of the ST segment and the end of the T wave; iii) presence of interpolated extrasystoles, compromising the measurement of the RR interval used to calculate the QTc interval; iv) ECG recordings with just one sinus beat per lead; and v) ECGs excluding more than three leads.

**Figure 1:**
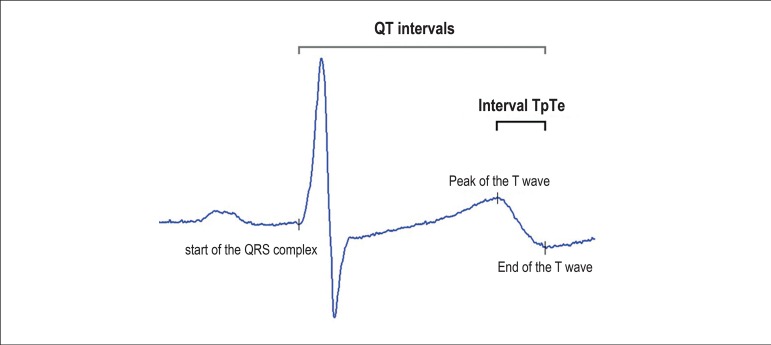
Illustration of the measurements of the QT intervals (interval between the start of the QRS complex and the end of the T wave) and TpTe (interval between the peak and the end of the T wave) in a surface electrocardiogram lead.

The QT and TpTe intervals were measured in all leads. We defined as isoelectric the line drawn between the PR interval of one beat and the TP interval immediately after the corresponding T wave. The end of the T wave was defined as the intersection between the largest tangent of the terminal phase of the T wave and the isoelectric line.^13^ The T wave peak was measured at the greatest vertical amplitude of the T wave relative to the isoelectric line. When the U wave was present, the end point of the T wave was measured as the nadir between the T and U waves. When the amplitude of the T wave was < 1.5 mm in a lead, this lead was excluded from the analysis. The QT interval was measured as the distance between the start of the first deflection relative to the QRS complex and the end of the T wave. In each lead, the QT and the TpTe intervals were calculated as the mean of three distinct beats. For each beat analyzed, we performed the measurement of the immediately preceding RR interval for QT interval correction, using the Bazett formula.^14^ The dispersions of the QT (DQT) and QTc (DQTc) intervals were calculated as the difference between the respective maximum and minimum values in the 12 leads. Thus, we calculated, in general, 36 QT, QTc, and TpTe intervals for each ECG. We considered as normal those values for the QT interval ≤ 0.44 s for men and ≤ 0.46 s for women after puberty. A DQT ≤ 0.06 s was considered normal. An external expert, oblivious to the clinical status of the study subjects, evaluated the electrocardiographic tracings. Figure 1 illustrates the means of the QT and TpTe intervals from one ECG lead.

We conducted paired Student's *t* and Bland-Altman tests to assess the reproducibility of the measurements from the TpTe interval. The reason for us to carry out such an assessment was that the peak of the T wave has an oval and almost flat appearance at the top, which could influence the value measured. Regarding the QT interval, the reproducibility of this variable is well known and, in the worst case scenario, the TpTe reproducibility assessment infers the reproducibility of the first. For each ECG lead, the same observer performed three measurements of the QT and TpTe intervals. The observer was asked to reposition the ruler after each measurement. Comparisons were made between the first and second, and between the second and third measurements for each interval. Of 2304 measurements of the TpTe interval carried out every run, a total of 1695, 1443, and 1443 in the first, second and third measurements, respectively, met the quality criteria and were included in the study. The differences between the first and second measurements, and between the second and third measurements were, respectively, 0.3 ms ± 12 ms (p = 0.59) and 0.3 ms ± 11 ms (p = 0.55). The Bland-Altman test revealed differences > 1.96 standard deviations (SD) in 1.7% of the measurements between the first and second assessments and 1.8% between the second and third assessments.

### Statistical analysis

Continuous variables are expressed as mean ± SD and nominal variables are expressed as absolute values ​​or frequencies (percentages). The groups were compared by Kruskal-Wallis one-way ANOVA for continuous variables and contingency tables (chi-square or Fisher's exact test) for categorical variables, where appropriate. The Fisher's test for distribution symmetry assessed normality. The Levine's test assessed the homoscedasticity between the groups. Receiver operating characteristic (ROC) curves were analyzed to assess the diagnostic capacity of each variable and identify optimal values of normality for the TpTe. The ROC curve area under the curve (AUC) test was done using the variable z. Cox's univariate and multivariate proportional hazard models were used to assess the prognostic value as regards the risk of events for the variables involved. Considering the number of observed endpoints, the multivariate survival analysis was performed only on the composite endpoints of death and transplantation in order to avoid overfitting. Only variables with significant beta coefficients in the univariate analysis were admitted in the multivariate model. The assessment of the adjustment of the data to the multivariate model was conducted by the Wald test. The proportional hazards assumption was tested by the correlation analysis (rho variable) between weighted Schoenfeld residues and survival time. The assumption of proportionality was accepted when the rho was not significantly different from zero. In the Cox models, the risk assigned to the Child variable was studied between the Child C group and the Child A and B groups aggregated (Child A+B). The statistical calculations were performed and the tables prepared with the programs R version 3.2.3 (R Core Team, 2015 Foundation for Statistical Computing, Vienna, Austria) and Medcalc 11.3.3.0 (MedCalc Software bvba, Belgium). The level of statistical significance adopted was 5%.

## Results

The demographic and clinical characteristics of the study subjects according to the Child classification at admission are shown in Table 1.

**Table 1:** Clinical, epidemiological, and laboratory characteristics of cirrhotic patients according to the Child-Pugh-Turcotte criteria

Variables*	Cirrhotic (N = 67)	Child A (N=25)	Child B (N=26)	Child C (N=16)
Age (years)	54.0 ± 12.9	55.2 ± 12.6	53.4 ± 38.6	53.2 ± 9.7
Male sex (%)	35 (52.2%)	13 (52%)	16 (61.5%)	8 (50%)
**Cirrhosis etiology**				
Alcohol (%)	A: 16 (23.9%)	A: 5 (20%)	A: 6 (23.1%)	A: 4 (25%)
Viral (%)	I: 27 (40.3%)	I: 8 (32%)	I: 9 (34.6%)	I: 8 (50%)
Alcohol/viral (%)	M: 4 (6.0%)	M: 3 (12%)	M: 3 (11.5%)	M: 0 (0%)
Other (%)	O: 20 (29.9%)	O: 9 (36%)	O: 8 (30.8%)	O: 4 (25%)
Beta-blocker (%)	30 (44.8%)	0 (0%)	14 (53.8%)	16 (100%)
Ascites (%)	38 (52.2%)	2 (8%)	17 (65.4%)	16 (88.9%)
Total serum bilirubin (mg/dL)	2.1 ± 2.0	0.9 ± 0.5	2.2 ± 2.0	3.8 ± 2.3
Serum albumin (g/dL)	3.3 ± 0.7	3.8 ± 0.6	3.1 ± 0.6	2.8 ± 0.7
INR	1.3 ± 0.3	1.2 ± 0.2	1.3 ± 0.2	1.6 ± 0.4
Serum creatinine (mg/dL)	0.9 ± 0.4	0.9 ± 0.6	0.9 ± 0.2	0.9 ± 0.4
Hematocrit (%)	35.6 ± 5.1	37.4 ± 4.9	35.2 ± 4.5	32.8 ± 5.4
Plasma renin activity (ng/mL/h)	7.0 ± 9.7	3.0 ± 2.5	8.3 ± 10.0	13.0 ± 14.8
Plasma norepinephrine (pg/mL)	196.2 ± 134.9	143.9 ± 106.0	220.8 ± 132.1	258.1 ± 164.2
Troponin levels (ng/mL)	0.05 ± 0	0.05 ± 0	0.05 ± 0	0.05 ± 0
BNP (pg/mL)	38.1 ± 42.3	28.7 ± 41.2	40.5 ± 34.2	54.6 ± 57.0
Liver transplantations	9 (13.4%)	0 (0.0%)	6 (23.1%)	3 (18.8%)
Deaths	12 (17.9%)	3 (12.0%)	4 (15.4%)	5 (31.3%)

* Continuous variables: Mean ± SD; categorical variables: absolute value, percentages between parentheses. INR: international normalized ratio; BNP: B-type natriuretic peptide.

After a median clinical follow-up of 12 months (4-13 months), corresponding to 659 patients/month, nine subjects underwent liver transplantation (Child A: 0 [0%], Child B: 6 [23.1%], Child C: 3 [18.8%], p = 0.04) and 12 died (Child A: 3 [12.0%], Child B: 4 [15.4%], Child C: 5 [31.3%], p = 0.002). The overall transplant and death rates were, respectively, 2.9% per year and 2.6% per year. The aggregate transplant and death rate was 3.2% per year. During follow-up, the occurrence of the composite endpoint of death and liver transplantation was higher in the Child C group than in groups A and B (Child A: 10.0%, Child B: 35.5%, Child C: 50.0%, p = 0.008, p linear trend = 0.002).

Of 79 ECGs analyzed at rest, 12 (15.2%) were excluded due to inadequate quality (Child A: 5, Child B: 5, Child C: 2). Thus, 67 electrocardiographic tracings were analyzed (Child A: 25, Child B: 26, Child C: 16).

The Fisher (for distribution symmetry) and Levine tests confirmed, respectively, the normal distribution of the variables and homoscedasticity among the groups analyzed, in the established alpha error limits. There was no difference between the cirrhotic groups regarding the QT, QTc and TpTe intervals, and DQT and DQTc (Table 2). The percentages of abnormal QT and QTc intervals, and DQT and DQTc were, respectively, 8%, 20%, 16%, and 24% in the Child A group; 7.4%, 22.2%, 14.8%, and 22.2% in the Child B group; and 0%, 25.0%, 0%, and 6.3% in the Child C group (Child A, B and C intergroup comparisons: p = 0.52, p = 0.77, p = 0.39, and p = 0.38, respectively).

**Table 2:** Characteristics of the variables related to ventricular repolarization in cirrhotic subjects, evaluated according to disease severity and clinical outcome

Variable	Child A (N: 25)	Child B (N: 26)	Child C (N: 16)	p*	Survivors (N=)	Death/transp (N=)	p†
RR interval (ms)	922 ± 138	955 ± 130	866 ± 141	0.12	922 ± 135	911 ± 134	0.69
Maximum QT (ms)	413.6 ± 32.1	425.3 ± 27.3	413.3 ± 32.9	0.40	419.0 ± 32.4	417.9 ± 26.3	0.96
Minimum QT (ms)	369.7 ± 31.5	383.8 ± 29	376.5 ± 30.7	0.34	376.7 ± 30.6	376.8 ± 26.5	0.99
Mean QT (ms)	394.2 ± 32.7	407.1 ± 25.3	395.3 ± 31.8	0.34	399.8 ± 32.2	400.5 ± 23.7	0.99
QT > 460 ms (%)	8.0%	7.4%	0.0%	0.52	8.2%	0.0%	0.48
DQT (ms)	43.9 ± 17.7	41.5 ± 21.9	36.9 ± 9.9	0.52	41.8 ± 19.2	41.1 ± 14.9	0.91
DQT > 60 ms (%)	16.0%	14.8%	0.0%	0.39	6.1%	10.5%	0.92
Maximum QTc (ms)	447.5 ± 72.7	442.1 ± 23.6	447.6 ± 22.9	0.91	446.9 ± 54.4	441.6 ± 20.9	0.54
Minimum QTc (ms)	387.7 ± 30.8	393.2 ± 31.5	403.9 ± 22.8	0.28	393.1 ± 31.7	395.3 ± 25.7	0.97
Mean QTc (ms)	414.6 ± 33.8	418 ± 20.9	426.4 ± 21.8	0.45	417.8 ± 27.9	421.6 ± 22.9	0.73
QTc > 460ms	20.0%	22.2%	25.0%	0.77	22.4%	10.5%	0.44
DQTc (ms)	59.8 ± 59.7	48.8 ± 22.9	43.7 ± 12.1	0.44	53.5 ± 44.8	46.8 ± 16.0	0.36
DQTc > 60 ms	24.0%	22.2%	6.3%	0.38	18.4%	15.8%	0.92
Maximum TpTe (ms)	87.2 ± 17.3	85.3 ± 11.4	82 ± 12.3	0.36	86.3 ± 15.1	78.4 ± 9.6	0.015
Minimum TpTe (ms)	54.3 ± 8.5	50.8 ± 8.3	56.1 ± 10.7	0.23	54.7 ± 9.4	50.5 ± 7.1	0.028
Mean TpTe (ms)	70 ± 6.9	67.9 ± 6.7	71.1 ± 8.8	0.45	70.8 ± 7.3	66.8 ± 8.2	0.029
TpTe ≤ 50 ms‡	32.0%	51.9%	37.5%	0.50	40.8%	73.7%	0.03

* One-way ANOVA; unpaired Student’s t test;

‡ optimal cutoff value calculated after analysis of the respective ROC; data are represented as mean

± SD or percentage; TpTe: interval between the peak and the end of the T wave; QT: QT interval; QTc: corrected QT interval; DQT: QT interval dispersion; DQTc: QTc interval dispersion; Child: Child-Pugh-Turcotte classification; Transp: liver transplantation (see text for details).

The ROC curves showed that only medium TpTe ≤ 60 ms and minimum TpTe ≤ 50 ms were predictors of death, with a sensitivity of 60% and 90% and a specificity of 79.3% and 57%, respectively (AUC = 0.76, p = 0.03, and AUC = 0.69, p = 0.006, respectively). Similarly, the maximum TpTe ≤ 80 ms and minimum TpTe ≤ 50 ms were composite endpoint predictors of death and transplantation, with a sensitivity of 84% and 73% and a specificity of 33% and 59%, respectively (AUC = 0.64, p = 0.04, and AUC = 0.64, p = 0.03, respectively). Considering the TpTe interval values of ≤ 50 ms as indicators of abnormality, the corresponding percentages in each group were 32% for Child A, 51.9% for Child B and 35.7% for Child C (p = 0.50 for comparisons between the Child A, B, and C groups).

Survival analysis as per the Cox proportional model adjusted well to the data and showed that the only predictors of death were the minimum TpTe interval of ≤ 50 ms (hazard ratio [HR] 6.5, 95% confidence interval [95%CI] 1.4 - 30.6, p = 0.02) and the medium TpTe interval of ≤ 60 ms (HR 4.6, 95%CI 1.3 - 16.2, p = 0.02) (Table 3). In relation to the composite endpoint of death and transplantation, the only predictors were Child group variables (Child C versus Child A + B, HR 6.3, 95%CI 1.5 - 27.2, p = 0.01) and the minimum TpTe interval of ≤ 50 ms (HR 3.9, 95%CI 1.5 - 10.4, p = 0.006) (Table 3). The Child group was not a predictor of death, and the TpTe maximum of ≤ 80 ms was not a composite endpoint predictor of death and liver transplantation.

**Table 3:** Cox proportional hazards univariate and multivariate analyses of the variables.

	Univariate Analysis						Multivariate Analysis		
**Variables**	**Death outcome**			**Composite outcome**			**Composite outcome**		
	**Beta**	**HR**	**95%CI**	**Beta**	**HR**	**95%CI**	**Beta**	**HR**	**95%CI**
Child group†	1.0	2.7	0.6-12.4	1.8*	6.3	1.5-27.2	1.4*	4.1	1.6-10.3
Minimum TpTe ≤ 50ms	1.9*	6.5	1.4-30.6	1.4*	3.9	1.5-10.4	1.2*	3.4	1.4-8.6
Mean TpTe ≤ 60 ms	1.5*	4.6	1.3-16.2	0.9	2.4	0.9-6.7	-		
Maximum TpTe ≤ 80 ms	-0.6	0.5	0.1-2.5	-1.0	0.4	0.1-1.3	-		

* p < 0.05;

† Child C versus Child A+B; multivariate analysis: Wald test *χ*^2^ 2df = 15.68, p = 0.0004;

Schoenfeld residual correlation test: Child group: rho = -0.21, p = 0.40; minimum TpTe ≤ 50ms: rho= rho=0.03; p = 0.91; beta: Cox proportional hazards model beta coefficient; HR: hazard ratio; 95%CI: 95% confidence interval; df: degrees of freedom; rho: correlation coefficient (see text for details).

**Figure 2:**
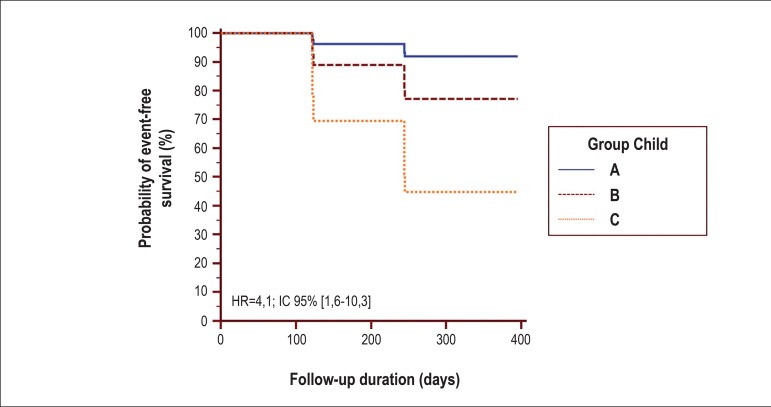
Kaplan-Meier curves adjusted to the multivariate model of Cox proportional hazards for the groups Child A, B, and C on the composite outcome death and liver transplantation. Risk ratio between Child [C] and Child [A+B] (HR = 4.1, 95%CI 1.6–10.3, p = 0.003). HR: hazard ratio, 95%CI: 95% confidence interval (see Table 3 and text for details).

**Figure 3:**
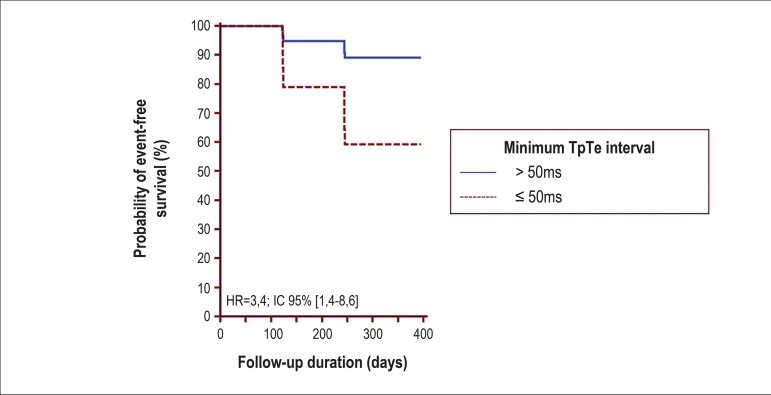
Kaplan-Meier curves adjusted to the multivariate model of Cox proportional hazards for the minimum TpTe interval related to the composite outcome death and liver transplantation (HR = 3.4, 95%CI 1.4 – 8.6, p = 0.008). HR: hazard ratio, 95%CI: 95% confidence interval (see Table 3 and text for details).

The multivariate analysis revealed that only the minimum TpTe interval of ≤ 50 ms along with the Child group was an independent composite endpoint predictor of death and transplantation (Table 3, Figures 2 and 3).

## Discussion

The Child-Pugh-Turcotte classification is a classic index of mortality in cirrhotic patients, initially used to define the prognosis of these patients when undergoing surgery for treatment of portal hypertension and today used as a reference prognosis for cirrhotic patients in general.^15^ Despite the creation of different prognostic rates in cirrhosis over the past decades, a systematic review of the European Association for the Study of the Liver (EASL), which assessed the natural history of cirrhosis as well as its prognostic indicators, concluded that the Child-Pugh-Turcotte classification was the predictive factor for independent mortality found more frequently in 67 studies including multivariate analyses of mortality predictors.^16^

In a consecutive series of cirrhotic patients conducted in a reference center for the treatment of liver diseases, the current study showed that the variables of ventricular repolarization - more specifically, QT, QTc, TpTe, DQT, DQTc - did not present significant variations related to the severity of the disease. This fact is clinically relevant since the increase in the QT interval may be related to malignant arrhythmogenic events and, therefore, to a reserved prognosis.^17^

Several experimental studies in cirrhotic patients have demonstrated that cardiovascular impairment is not restricted only to hemodynamic changes in peripheral circulation. Several pathophysiological mechanisms have been proposed to justify the changes observed in the heart of cirrhotic patients: blocking of the beta-adrenergic receptor, increase in fluidity of the cell plasma membrane by the composition of different lipids, exposure of cardiomyocytes to cardiodepressant substances, changes in the dynamics of intracellular calcium, and structural and functional changes in potassium channels, among others.^18^

The association between the severity of the liver disease and cardiac impairment is not entirely clear. Some studies suggest that the QT and QTc intervals correlate with the Child-Pugh-Turcotte class and that both are prognostic markers in cirrhotic cardiomyopathy.^5^

Hendricksse et al. observed evidence of vagal neuropathy associated with prolongation of the QTc interval in more than 50% of the patients with cirrhosis.^19^ Kempler et al. analyzed the QTc and indices of parasympathetic activity through various maneuvers (handgrip, Valsalva, deep breathing, and orthostatism) in 83 patients with alcoholic cirrhosis. These authors found, by linear regression, a significant correlation between the QTc and the degree of autonomic dysfunction, with a mean QTc value of 408 ms in patients with normal performance in autonomic function tests and 497 ms in those with abnormalities in all indices of autonomic dysfunction.^20^

The TpTe corresponds to the transmural dispersion of ventricular myocardial repolarization, a period in which the epicardial region is repolarized, and the M cells are still in the repolarization process, therefore vulnerable to the occurrence of early post-depolarization events.^21,22^ Elevated TpTe values have been associated with arrhythmic events in various clinical conditions and may, therefore, be a factor of poor prognosis in several clinical events. In particular, enlargement of the TpTe interval has been observed in acquired or congenital long QT syndrome, in hypertrophic cardiomyopathies with troponin mutation,^23^ and in patients undergoing primary percutaneous coronary intervention for acute myocardial infarction.^24^

In our study, we found no association between changes in electrocardiographic variables of ventricular repolarization and the severity of the cirrhotic disease. The QT and QTc intervals, whose values ​​are described in the literature as elevated according to the progression of the disease, showed no differences among the analyzed groups, and there was no difference between the groups regarding the QT and QTc values ​​above the normal range. We also did not observe differences in the values for the TpTe variables among the cirrhotic groups or in relation to the normal values. One possible explanation for this may have been the inclusion of a reduced number of patients with greater disease severity (Child C), as this group, due to its clinical features, has increased mortality and is quickly referred for transplantation or palliative procedures, such as implant of TIPS, which certainly contributed to impair the increased sample size of this group.

Regarding the prognostic value, we observed that the minimum, maximum, and mean TpTe were prognostic indicators of overall survival and transplant-free survival during the analyzed period, with a specificity of 79.3%, while the minimum TpTe below 50 ms was a poor prognostic indicator, with a sensitivity of 90%. These parameters can further serve as a reference to identify cirrhotic patient groups with the potential to evolve to death and, therefore, indicate a need for early intervention (transplantation). Whereas, in relation to the QT and its variables, we observed no prognostic impact on their values.

The literature shows that an increase in TpTe is more related to arrhythmic events due to the increased dispersion of transmural ventricular repolarization, which triggers substrates for reentry arrhythmia. In our study, we observed that a short TpTe, but not a long one, is significantly related to a higher rate of death/transplantation. One possible explanation for this finding is that a short TpTe would be a sign of severe disease in cirrhotic patients and an electrical characteristic in this group of patients. We speculate that several causes may contribute to this finding, such as changes of repolarization in the membrane of cardiomyocytes due to cardiodepressant substances that accumulate in cirrhotic patients, in addition to changes in the dynamics of intracellular calcium and potassium channels.

According to our knowledge, this study was the first to demonstrate a relationship between a low TpTe and a poor prognosis in cirrhotic patients, which can lead to a major change in behavior (early transplantation) in patients who have this indicator. We believe, with the continuation of the study and development of new ones, that the relationship between this new electrocardiographic marker of ventricular repolarization and the survival of patients will be better assessed, and their true clinical role will be established.

### Limitations

The limitations of the present study are: 1) the inclusion of a convenience sample, although it was recruited from a specialized SUS center; 2) the difficulty of locating the peak and the end of the T wave when it is biphasic or has a more flattened appearance, which could affect both the QT interval and the TpTe measurements, although the reproducibility analysis has shown that the measurement was satisfactory; 3) the description in the literature of the TpTe interval as a marker of adverse outcomes. In this study, lower values of the TpTe interval have shown to be an independent prognostic marker of adverse events in the population of patients with liver cirrhosis. In other populations, the TpTe interval has shown values above certain limits of normality. No electrophysiological information about the behavior of the transmural dispersion of repolarization in patients with cirrhotic cardiopathy was available in the literature up to the time when this study was conducted. Although objects of speculation, these observations indicate that in cirrhotic patients, the transmural gradient of ventricular repolarization, represented by the TpTe interval, is strongly attenuated as a result of an electrotonic cancellation, possibly influenced by the humoral environment related to the cirrhotic disease. Indeed, it was found that patients classified as having more severe cirrhotic disease had percentage values below 50 ms more frequently than those classified as having less severe cirrhotic disease. The impact of the blood ammonia concentration and other metabolites, and of the pH on the transmural refractory behavior, need to be investigated. The question is still open and needs to be confirmed in future studies; 4) it was not possible to relate the cause of death (arrhythmic *versus* non-arrhythmic) with the duration of the TpTe interval. Thus, it is possible that the TpTe interval behaved as a bystander or, in other words, it was obtained randomly in this present sample and not by causality. Further studies are needed to confirm these findings; 5) it was not possible to interrupt the use of beta-blockers since discontinuation might precipitate the occurrence of gastrointestinal bleeding in esophageal varices. Therefore, for ethical reasons, we chose to maintain the medication. Beta-blockers have several cardiac effects, including a reduction in basal heart rate, which could potentially affect the measurements of the QT and the TpTe intervals; 6) the measurements were performed by a specialist, who obtained three non-sequential measurements of the same lead. Therefore, there was control of intraobserver variability. Despite the low intraobserver variability, we agree with the possibility of a possible measurement bias; 7) this study was developed to assess the impact of ventricular function on the survival of patients with cirrhosis. Therefore, the sample measured and the study of electrocardiographic variables were natural consequences of its development. Thus, considering the severity of the cirrhotic disease itself, we believed it was unnecessary to use a healthy control group; therefore, we used the group with Child A classification as the control group.

## Conclusion

In this prospective analysis of cirrhotic patients classified according to the Child-Pugh-Turcotte criteria, the QT, QTc and TpTe intervals, and the DQT and DQTc did not correlate with disease severity. TpTe interval values of ≤ 50 ms and disease severity, according to the Child-Pugh-Turcotte criteria are independent prognostic markers of death and/or liver transplantation in this population.
